# Ecological Risks in Daqu Storage and Their Impact on Baijiu Flavor: Precision Process Strategies for Damage Mitigation While Preserving Aroma

**DOI:** 10.3390/foods15071195

**Published:** 2026-04-02

**Authors:** Dandan Song, Chunlin Zhang, Yashuai Wu, Liang Yang

**Affiliations:** 1 School of Brewing Engineering, Moutai Institute, Renhuai 564501, China; songdd0330@163.com (D.S.); zcl818075@163.com (C.Z.); 2 Guizhou Key Laboratory of Microbial Resources Exploration in Fermentation Industry, Kweichow Moutai Group, Zunyi 564501, China; 3 School of Food Science and Engineering, South China University of Technology, Guangzhou 510640, China; wyss995418706@163.com

**Keywords:** Daqu microecology, Baijiu fermentation, flavor formation, microbial community, process control

## Abstract

Daqu functions as a core saccharifying and fermenting starter in Baijiu production and acts as a complex microecological reactor in open solid-state fermentation. The formation of Baijiu flavor is closely associated with microbial community assembly, enzymatic activities, and metabolic interactions occurring within Daqu. However, the open production environment also exposes the Daqu system to multiple external disturbances that may influence its microecological stability and fermentation performance. This review summarizes recent advances in understanding the microecology of Daqu, focusing on microbial succession, metabolic pathways related to flavor formation, and environmental factors affecting Daqu quality. Particular attention is given to how external disturbances during production and storage may influence microbial communities, enzymatic functions, and aroma compound formation. Based on current knowledge, a conceptual framework linking environmental factors, microbial community dynamics, metabolic activity, and flavor outcomes is proposed. In addition, strategies for maintaining microecological stability and ensuring flavor consistency are discussed, including environmental management, process optimization, physical control technologies, and integrated quality monitoring systems. Emphasis is placed on combining process control with modern analytical approaches such as multi-omics technologies and process analytical technologies (PAT) to improve traceability and precision management during Daqu production. Overall, this review provides a systematic perspective on the relationships among Daqu microecology, process conditions, and flavor formation and highlights future research directions for achieving stable and controllable Baijiu fermentation systems.

## 1. Introduction

Baijiu was one of the six major distilled spirits worldwide and was distinguished by a traditional process in which solid raw materials underwent simultaneous saccharification and fermentation [1,2,3,4]. In this process, Daqu served as the core saccharifying and fermenting agent, and its quality directly determined brewing success and the flavor profile. Daqu production relied on open natural fermentation. Long domestication enriched a relatively stable and complex microbiota that provided diverse enzyme systems and flavor precursors and laid the foundation for Baijiu fermentation [5,6]. Studies showed that bacteria, fungi (including molds and yeasts), and actinomycetes commonly coexisted in Daqu and together formed a dynamically balanced microecosystem. This unique microbial ecology endowed Baijiu with complex and diverse flavors and a distinctive aroma spectrum [7,8]. The openness that created flavor diversity also made stability susceptible to external influences. Invasion by exogenous microbes or insects could disrupt the balance. During production and storage, undesirable microbes unavoidably mixed into the system and caused fluctuations in Daqu quality, reduced alcohol yield, and increased costs [9,10,11].

In recent years, particular attention has been drawn to Daqu-associated pests. During cultivation and storage, Daqu was prone to infestation by various stored product insects, among which *Tribolium castaneum* and the coffee bean weevil were dominant grain pests [12]. These pests multiplied within Daqu and consumed and bored through bricks, which led to the loss of active components. Their activity also altered internal distributions of moisture and nutrients and affected physicochemical properties and enzyme activities. Reports indicated that severe infestation caused marked declines in saccharifying and fermentative power, with quality losses up to 20% to 30%, and serious deterioration of aroma [9]. More concealed interactions likely existed between pests and the Daqu microbiota [9,13,14]. On one hand, pests carried or enriched certain microbes from the environment, and their feeding could alter community structure. On the other hand, symbionts in the insect gut overlapped with fungi in Daqu and suggested a vector role during feeding that influenced microbial distribution within bricks. These findings prompted a reassessment of the ecological role of pests and highlighted a special challenge in maintaining microecological stability and flavor.

To ensure flavor consistency and process controllability, both the complexity and stability of the Daqu microecosystem required attention [15,16,17,18]. The internal interactions of the microbiota and their impact on flavor synthesis needed to be resolved with modern microbiome approaches [19,20]. External disturbance by pests also needs effective process control [21]. In traditional practice, functional strains or consortia had been added to adjust the community and enhance enzyme activity and aroma formation. Because of intrinsic complexity, such reinforcement sometimes failed to meet expectations and could cause flavor imbalance or quality decline [22]. Pest management faced a similar dilemma. Heavy reliance on chemical insecticides could damage beneficial microbes and raise residue concerns, while a lack of control allowed pests to impair flavor [23,24]. How to balance microecology, flavor, and process control and achieve precise pest control while maintaining normal fermentation and aroma formation has become an urgent challenge for the industry.

Encouraging progress had been reported. Low-dose electron beam irradiation was explored to disinfect Daqu while essentially preserving the microbiota [12]. At suitable doses, treated Daqu recovered its microecology to untreated levels after months of storage, and key indices such as saccharifying power, liquefying power, and acidity met brewing requirements [25,26,27]. This suggested a path to control pests without constraining aroma by removing insects with precise measures while protecting aroma formation. Researchers also proposed RNA interference to target pest symbionts for green control [9,28,29]. These directions reflected the integration of ecological mechanisms and process practice. They clarified how infestation affected ecology and offered strategies that maintained disinfestation efficacy and protected flavor.

In summary, Daqu played an irreplaceable role in Baijiu brewing. The impacts of pests on flavor and Daqu quality and the resulting fluctuations and instability urged closer examination of the interaction among microecology, flavor, and control. With advances in ecological fermentation and community regulation, strengthening research on the Daqu microbiome and developing precise pest control technologies were expected to stabilize flavor and optimize traditional practice. This review aims to summarize recent progress on Daqu microecology, flavor formation, and pest control and to explore the path from ecological mechanisms to process implementation that controls pests without constraining aroma, providing new ideas for quality control in Baijiu brewing.

To improve the transparency and reproducibility of this review, the relevant literature was retrieved primarily from the Web of Science database. The search covered publications indexed from the inception of the database to 28 February 2026. A keyword-based search strategy was adopted, and the search terms were developed on the basis of the title, abstract, and keywords of the present review, including Daqu, Baijiu fermentation, Daqu microecology, flavor formation, microbial community, process control, ecological risks, environmental disturbances, Daqu storage, aroma preservation, damage mitigation, and solid-state fermentation. The retrieved studies were then screened according to their relevance to Daqu microecology, flavor formation, environmental disturbance, storage risk, and process control during Daqu production and storage.

## 2. Daqu Production Processes and the Microenvironments That Enabled Colonization by Daqu-Associated Pests

### 2.1. Definition, Classification, and Core Functions of Daqu

Daqu was a solid saccharifying and fermenting agent made mainly from wheat or from wheat with barley or peas. It was obtained through open natural fermentation, and it provided enzymes, starter, and aroma [30,31]. In essence, it was a porous, brick-like microhabitat in which self-heating occurred in the qu room. Microbial metabolism generated biothermal energy and moisture gradients. These forces selected functional consortia with distinct tolerance and metabolic traits. The consortia supplied diverse enzyme systems and flavor precursors for saccharification, seed mash establishment, and main fermentation. Prior studies treated Daqu as a shared ecological reactor in solid-state fermentation. Coupled biothermal flux and humidity with oxygen loss were shown to drive community assembly. This explained the marked differences in communities and metabolic trajectories among sites and seasons [32,33].

Based on the peak temperature reached during making, Daqu was grouped into three types. High-temperature Daqu reached about sixty to seventy degrees Celsius. Medium-temperature Daqu reached about fifty to sixty degrees Celsius. Low-temperature Daqu reached about forty to fifty degrees Celsius [30]. Each range favored different dominant groups and enzyme profiles and thus shaped flavor styles. High-temperature Daqu tended to enrich thermotolerant bacilli and actinomycetes and supported precursors for roasted and sauce-like notes. Medium-temperature Daqu balanced saccharification and ester formation. Low-temperature Daqu retained more yeasts and *Aspergillus* for saccharification and aroma and was linked to softer profiles. Evidence for the temperature ranges and the linkage between flavor and function had recurred across comparative studies and had been verified on multiple Daqu types [34,35,36].

The high-temperature stage reshaped not only enzyme patterns but also networks of flavor precursors. In sauce-aroma production, high-temperature Daqu often reached about seventy degrees Celsius. The heat reduced the expression of some saccharification enzymes yet raised pathways for the degradation of aromatic compounds and for building roasted precursors. This suggested a tradeoff in which saccharification was reduced while precursors were promoted. Such thresholds explained divergent aroma backbones during later stacked fermentation under different temperature regimes [37]. Daqu was not only a collection of functional microbes. It was also a microecological mosaic molded by the open environment and grain substrates. The process drew exogenous microbes from air, tools, and raw materials. Temperature and humidity curves and ventilation strategies in the qu room created spatial heterogeneity across positions. A combined passive domestication and active selection produced a mature community and metabolic fingerprint distinct from the initial state [26,38]. This natural selection effect was observable at metagenomic and metabolic phenotypes and was tightly linked to internal and surface gradients [26,38,39,40].

From the process perspective, four core functions were evident [41,42]. First, enzyme supply. Complex enzyme systems such as amylases, proteases, lipases, and polysaccharide-degrading enzymes provided fermentable substrates and flavor precursors. Second, starter supply. Relay communities of bacteria, yeasts, and filamentous fungi seeded stacked and pit fermentations [43,44]. Third, aroma formation. Heat stress and redox fluctuations in the bricks promoted pyrazines, carbonyls, and many ester precursors. Fourth, regulation. Physical and chemical indices and microbial quality of Daqu shaped community structure during main fermentation and the flavor of the final product. Fluctuations in Daqu quality translated into differences in yield and aroma spectra [45,46].

These traits meant that Daqu carried a compound microenvironment of heat, moisture, matrix, and surfaces. This could overlap with the ecological niches of Daqu-associated pests. The insects fed on grain fines and fungal biofilms and preferred high humidity and habitable pores. Production, maturation, and storage offered a continuous environmental gradient. After a phase of lethal heat, cooling created suitable periods. These windows permitted occasional or seasonal colonization.

### 2.2. Process Flows of Different Daqu Types and Their Microenvironmental Features

A typical flow included raw material selection and milling, moistening and mixing, brick forming and pressing, room entry with stacked fermentation under controlled temperature and humidity, turning with slow cooling, discharge with air-drying, and maturation storage [45,47]. The timeline often spanned several weeks to more than one month. During stacking, the bricks self-heated to a peak plateau. Cooling and ripening then followed. Sites and aroma styles differed in ratios, moisture, stacking modes, and ventilation regimes. Yet the shared physical chain of self-heating, water migration, oxygen diffusion, and surface renewal was consistent. Practice and research showed that this chain governed community succession and built superposed temperature and humidity gradients in both the room and the bricks [6,7,48].

**Figure 1:**
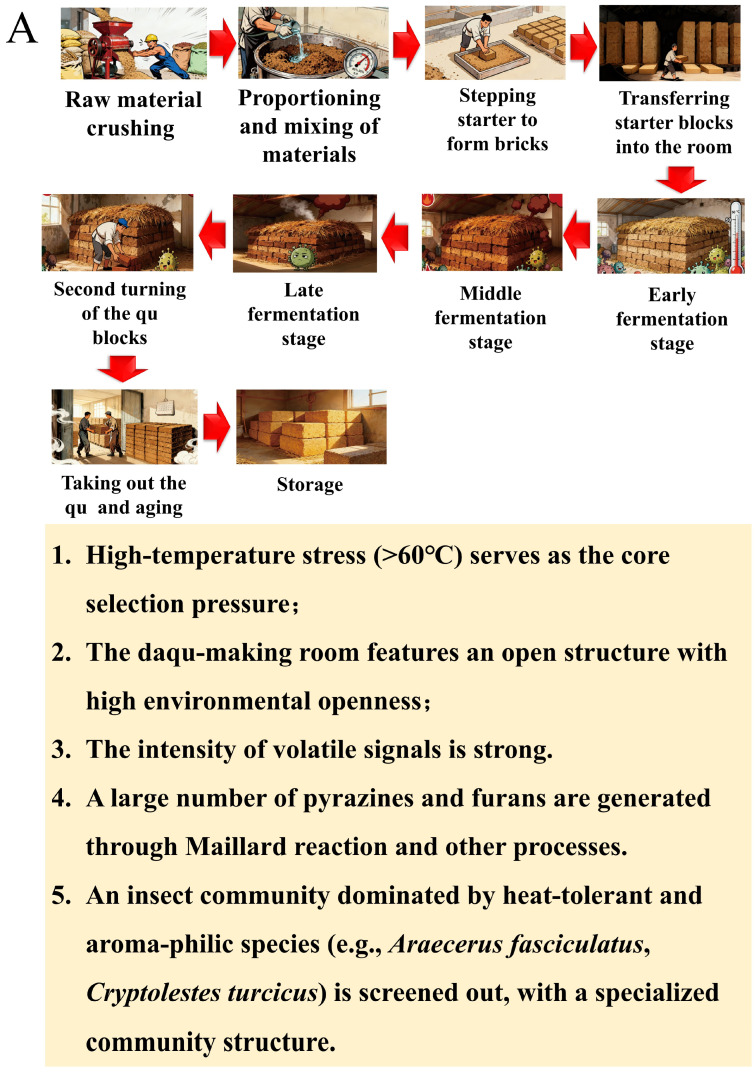

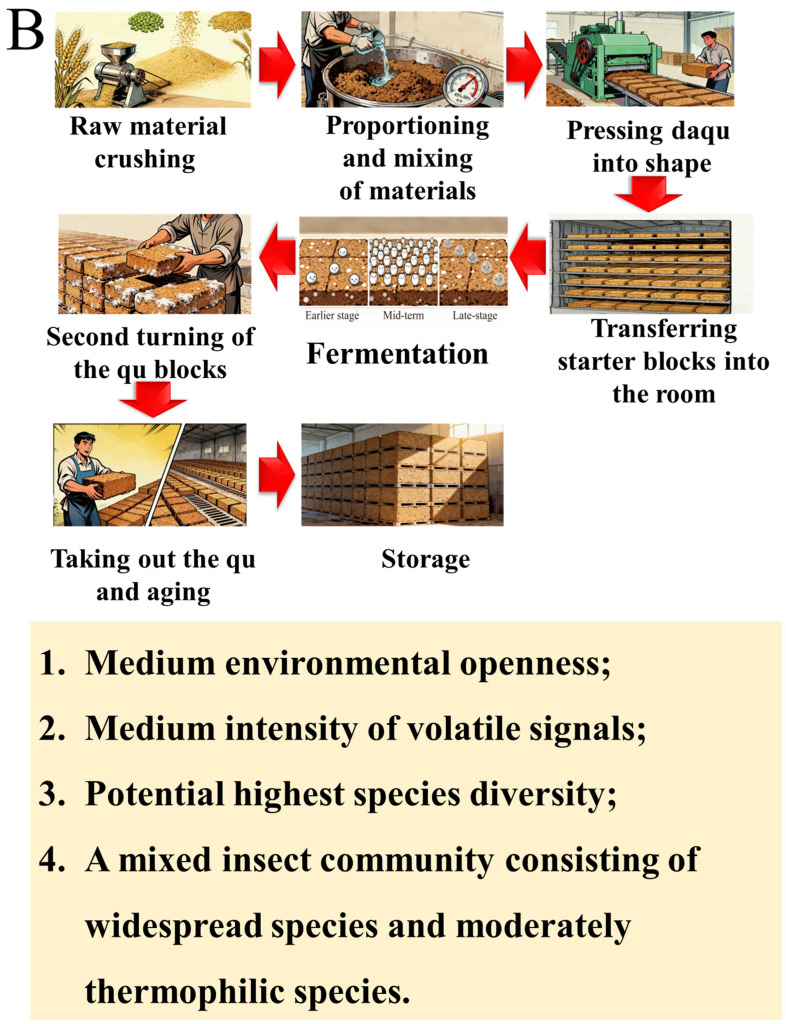

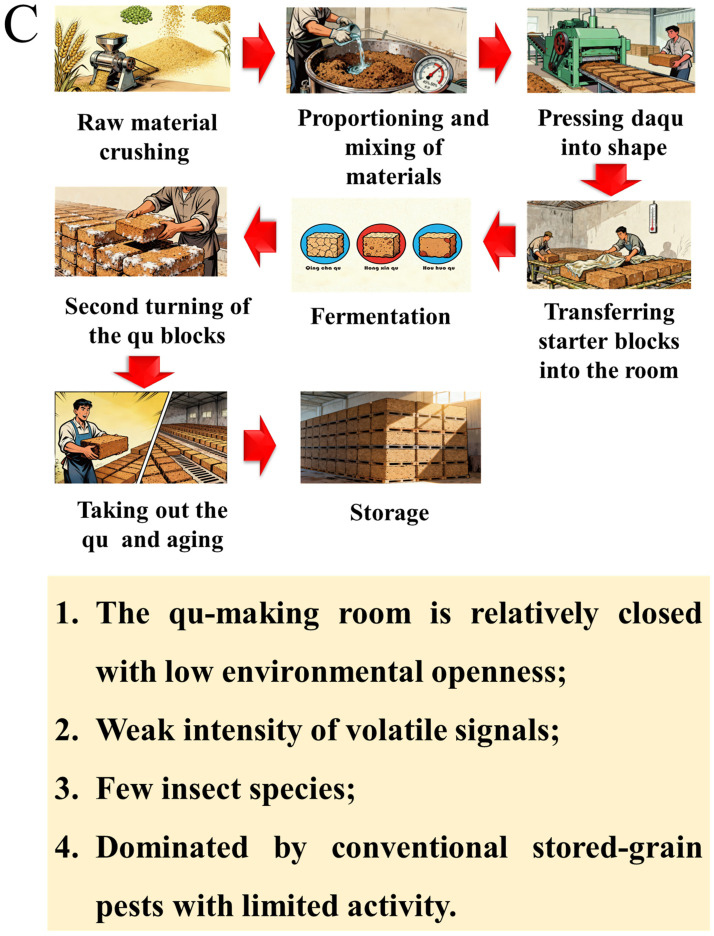
Production process flowchart of Daqu. (**A**) High-temperature Daqu; (**B**) medium-temperature Daqu; (**C**) low-temperature Daqu.

According to Figure 1A and Supplementary Table S1, high-temperature Daqu showed the strongest heat pulse. Continuous monitoring showed that about one week after room entry, the peak reached not less than sixty degrees Celsius and could persist for several days to more than a week. Internal relative humidity stayed high, and oxygen diffusion was restricted. The surface dried and exchanged gases by convection. The depth gradient allowed thermotolerant actinomycetes, *Bacillus*, and thermotolerant fungi to occupy different strata. Marked differences in communities and functional roles arose between the core and the surface. As the temperature fell, water redistributed and moved outward. Surface microbes regained an advantage. Mature Daqu thus displayed spatial heterogeneity [49,50,51].

Medium-temperature Daqu had milder temperature and humidity trajectories yet showed higher sensitivity to environmental management. Room temperature and relative humidity altered the heating rate and drying speed of the bricks and thereby shifted community composition and interaction networks. Room humidity strongly drove the succession of *Aspergillus*, *Wickerhamomyces*, and several filamentous fungi. Room temperature correlated more directly with the rise and fall of yeasts (Figure 1B, Supplementary Table S1). This indicated that coupling between process and microecology could be tuned by finer ventilation and turning schedules to balance saccharification, fermentation, and ester potential [20,52,53].

Low-temperature Daqu typically peaked at about forty-five to fifty degrees Celsius. Communities retained more *Aspergillus*, mildly thermotolerant fungi, and yeasts, and were often associated with gentle and clean aromas (Figure 1C, Supplementary Table S1). Because heat stress was weaker, enzyme fidelity and diversity in aromatic metabolism reflected a mild balance. Such microenvironments suited production aimed at elegant styles [7,54].

Within the same temperature class, molding and stacking altered porosity and channels for heat and moisture transfer. Mature bricks thus formed micro-landforms at different depths. The result appeared as spatial differences in aroma-active compounds and dominant taxa. Recent multi-batch comparisons showed that coupling among structure, microbes, and flavor explained differences in aroma among workshops in the same plant and among stacks in the same batch. This suggested that structural variables were manageable ecological variables [55,56,57].

Turning was a key traditional operation and acted as a microenvironment rebalancing step. It broke local overheating and anoxia, rebuilt convection paths and vapor escape, and brought surface-to-core coupling of temperature, humidity, and oxygen back into a controllable zone. In medium-temperature production, small changes in the timing and frequency of turning altered community assembly and metabolic phenotypes in the following days. Mechanistic interpretation had shifted from experience, such as preventing caking, to process optimization based on community dynamics and heat transfer models [58].

Slow air-drying after discharge and maturation were further micro-environment shaping stages that had been undervalued. As free water continued to decline and highly volatile components were removed, aroma stabilized. Off-odors were smoothed. The bricks still underwent slow metabolism and chemical reactions under microaerobic conditions. A more stable base of enzymes and aroma was thus built. This later ripening, together with the earlier heat shock, formed a two-stage setting of Daqu quality over time [6,30].

Viewed through the niche of pests, several windows for colonization were evident [10,59]. The early high-humidity phase after room entry and stacking offered free water and broken particles that supported insect feeding on biofilms and grain fines. The subsequent heat platform imposed lethal stress, especially at the core. During the cooling and drying transition, surfaces and crevices with temperatures around twenty to thirty degrees Celsius and still high humidity became key refuges. During maturation storage, high relative humidity in the warehouse and biofilm on the brick surface promoted expansion of populations in surfaces, pores, and joints. Stored product insects and storage mites were highly sensitive to humidity. Rising critical relative humidity multiplied development and reproduction. Drying or higher vapor pressure deficit shortened survival. Extensive studies on grains and hard cheeses showed that booklice, flour mites, and flour beetles developed rapidly at thirty-two to forty degrees Celsius under high relative humidity, whereas many mobile stages died quickly when humidity fell or when temperature rose to about forty to forty-five degrees Celsius [29]. These rules arose from storage ecology yet were transferable to the grain-based, high-humidity, open-surface conditions of Daqu production, maturation, and storage and should be included in integrated control of production and warehousing [59]. Furthermore, microenvironments under different temperature classes determined spatial patterns of potential harm. In high-temperature Daqu, the core during the peak was almost uninhabitable. The surface remained warm and moist for a longer time after cooling. If ventilation and drying were insufficient, the surface became the first choice for colonization. In medium- and low-temperature Daqu, the cooling was slower. Suitable windows inside the bricks appeared earlier and lasted longer. Earlier turning, destacking, and moisture control were therefore needed to narrow these windows. Translating these insights into practice meant designing processes that minimized pest niches by coupling trajectories of temperature and humidity, brick structure, and storage climate [32,60].

Airborne microbes inside and outside the qu-room and regional climate also influenced the initial state of microecology through temperature and precipitation and interacted with potential colonization by pests [61,62]. Cross-region comparisons showed that changes in year and climate factors altered the base community during early fermentation and indirectly shifted aroma composition and stability of mature Daqu [63]. This indicated that control of temperature and humidity in the process and drying during storage were not only internal variables. They needed coordination with the seasonal climate at the production site to form an integrated design that linked the qu room, storage, and climate.

## 3. Operational Definition, Representative Taxa, and Infestation Assessment of Daqu-Associated Pests

In the context of Daqu production and storage, Daqu-associated pests may be operationally defined as stored-product insects that are repeatedly detected in Daqu-making workshops, Daqu storage environments, or closely connected grain-handling spaces, and that are capable of colonizing raw materials, brick surfaces, cracks, dust layers, fines, or fungal biofilms, thereby interfering with physicochemical stability, microbial community assembly, enzymatic functions, or flavor consistency [12,59]. From the perspective of stored-product entomology, these insects can be broadly divided into primary pests, which are able to attack intact kernels or relatively compact substrates, and secondary pests, which preferentially exploit broken kernels, powders, dust, and mold-associated residues [64]. This classification is particularly relevant to the Daqu system because the production process creates a continuum of ecological niches ranging from intact grain particles to cracked surfaces, dust-rich corners, porous brick matrices, and post-cooling moist fissures. Therefore, the insects that deserve primary attention in the Daqu scenario are not all stored-product insects, but rather those species that are repeatedly reported in association with grain-based fermentation environments and that show clear ecological compatibility with the thermal, structural, and nutritional conditions of Daqu production and storage.

Based on the currently available literature and the biological summary compiled in the present revision, the most representative taxa included *Rhyzopertha dominica*, *Sitophilus zeamais*, *Sitophilus oryzae*, *Tribolium castaneum*, *Araecerus fasciculatus*, *Cryptolestes ferrugineus*, *Latheticus oryzae*, and *Cryptolestes turcicus* [59,65]. These species were selected because they collectively covered the major ecological roles that were relevant to the Daqu environment. *R. dominica*, *S. zeamais*, and *S. oryzae* represented internal or primary grain-feeding pests with a strong capacity to invade relatively intact grain-derived substrates [66]. *T. castaneum* represented a typical secondary flour and dust feeder and has already been reported as a major pest infesting sauce-flavor Daqu [67]. *A. fasciculatus* represented a warm- and humid-adapted polyphagous species with a strong affinity for mold-rich and nutrient-diverse substrates. *C. ferrugineus*, *L. oryzae*, and *C. turcicus* represented crack-associated and fines-associated secondary pests that were especially relevant to fragmented materials, dust deposits, and crevice-rich storage microhabitats [68,69] (Table 1). In other words, these taxa did not simply constitute a species list. Rather, they represented a functional pest assemblage that corresponded to the major ecological vulnerability points of the Daqu system, including intact grain invasion, powder-associated colonization, crack exploitation, and persistence under warm and humid storage conditions [59].

Infestation severity in the Daqu system should not be assessed by insect presence alone. A more useful framework should combine direct entomological indicators, microenvironmental indicators, and process-linked response indicators. Direct entomological indicators may include pheromone or food-bait trap captures, light-trap captures where applicable, direct insect counts from surface and crevice sampling, and detection of frass, exuviae, carcasses, or perforated grain residues [70,71]. Microenvironmental indicators may include dust and fines accumulation, crack width and density, localized moisture retention, relative humidity in stacking zones, and visible fungal film development [12,64]. Process-linked response indicators may include changes in saccharifying activity, liquefying activity, acidification behavior, microbial community deviation, and shifts in key volatile compounds associated with flavor quality [59,65]. In this sense, infestation severity should be understood as a multi-dimensional ecological disturbance state rather than as a simple insect-count variable. This integrated assessment is consistent with stored-product monitoring principles, in which trap data, spatial hotspot analysis, and environmental context are interpreted together rather than independently. It also better fits the Daqu system, where insect activity, microbial succession, and flavor precursor transformation are tightly coupled.

It should also be emphasized that a unified quantitative standard for Daqu infestation severity has not yet been established. For this reason, the present review proposed a practical assessment logic in which pest intensity was characterized by species identity + spatial position + residue signal + process response. Such an approach may help distinguish between low-level incidental occurrence and biologically meaningful infestation. For example, occasional captures of secondary beetles in peripheral corridors may indicate external immigration only, whereas repeated detection of flour-feeding or crack-dwelling species together with increasing dust deposits, fungal films, and physicochemical deviation in bricks may indicate active colonization with real process implications. Accordingly, the present review argued that the operational definition of Daqu-associated pests should be tied not only to taxonomy but also to ecological role, habitat compatibility, and process relevance.

**Table 1:** Operational summary of representative Daqu-associated pests, their ecological roles, and suggested indicators for infestation assessment.

Species	Major Ecological Role	Typical Niche in the Daqu System	Key Biological Features Relevant to Daqu	Suggested Infestation Indicators	Potential Impact on Fermentation and Flavor	References
*Rhyzopertha dominica*	Primary/internal feeder	Grain-derived particles, compact substrate zones, inner brick pores or surrounding crevices	Strong boring capacity, broad host range, rapid spread under warm and humid conditions	Adult captures, bored particles, kernel perforation, fine powder generation, and internal damage signs	Structural disruption, increased powdering, altered water activity, potential shift in microbial assembly	[72,73]
*Sitophilus zeamais*	Primary/internal feeder	Moist grain-rich areas, early-stage bricks, storage-linked grain interfaces	Flight-capable, concealed larval development, strong performance in warm and humid conditions	Trap captures near intake and storage, perforated kernels, emergence holes, and localized grain residues	Hidden damage to substrate integrity, rapid colonization, and possible reduction in process stability	[74,75,76]
*Sitophilus oryzae*	Primary/internal feeder	Humid southern or coastal production environments, early-stage bricks with grain residues	Internal feeding life cycle, relatively high fecundity, strong compatibility with moist grain-derived substrates	Surface and cross-section inspection, emergence holes, frass particles, and trap captures	Changes in pore structure and gas diffusion, altered release of enzymatic and aroma precursors	[77,78]
*Tribolium castaneum*	Secondary flour and dust feeder	Brick surface cracks, dust-rich corners, equipment bases, powder deposits	Polyphagous, rapid development, high fecundity, strong migration ability, strong adaptation to fines and flour	Pheromone trap captures, dust-associated counts, frass and exuviae residues, and repeated captures in surface hotspots	Surface nutrient disturbance, biofilm restructuring, contamination risk, possible off-odor contribution	[67,79,80]
*Araecerus fasciculatus*	Warm-humid polyphagous secondary pest	Mold-rich surfaces, wet cracks, stack edges, poorly ventilated corners	Broad host range, strong association with humid and fungus-rich substrates, good dispersal ability	Trap captures in warm-humid zones, detection in mold-associated substrates, and residue accumulation	Damage to brick surface structure, enhancement of fungal–insect interaction, instability of aroma development	[81,82,83]
*Cryptolestes ferrugineus*	Secondary crack and fines feeder	Broken particles, fines, dusty fissures, edge zones with moisture retention	Rapid life cycle, strong response to temperature and humidity gradients, high mobility in fragmented substrates	Trap captures in warm stack edges, counts in dust layers, and frass and exuviae in cracks	Expansion from edge to interior, increased dust and fragmentation, indirect disturbance of microecology	[69,84,85]
*Latheticus oryzae*	Secondary fissure and powder-associated feeder	Warm storage crevices, dusty corners, fragmented grain or flour residues	Multiple generations, preference for warm and humid conditions, strong crack-associated survival	Direct counts in crevices, dust accumulation index, repeated captures in poorly cleaned zones	Persistent hidden infestation, increased powder load, possible fungal co-amplification	[59,86]
*Cryptolestes turcicus*	Secondary humid fines-associated feeder	Warm and humid cracks, broken grain interfaces, powder-rich storage microhabitats	Moisture-dependent development, rapid spread in humid conditions, strong crevice affinity	Trap captures in humid storage zones, counts in crack-associated fines, and residue build-up	Enhanced risk under wet storage, persistent low-visibility colonization, disturbance to microenvironmental stability	[87,88,89]

## 4. Daqu Production and Microecology

Daqu is not merely a fermentation starter but also a structured biological carrier in which matrix architecture, porosity, moisture migration, aeration, temperature gradients, and microbial succession jointly determine saccharification, liquefaction, esterification, and subsequent flavor formation [6,39,42]. In this system, the physical brick structure and the microecological environment are tightly coupled. Stable internal channels for ventilation and drying support enzyme initiation, surface-to-core differentiation, and orderly community assembly, whereas disruption of these features may alter both fermentation performance and flavor development.

Accordingly, pest occurrence in Daqu production should not be interpreted only as a sanitation problem. More importantly, it should be regarded as a disturbance factor capable of interfering with the structural and ecological basis on which Daqu functionality depends. In other words, pests may influence the carrier that supports early microbial colonization, enzymatic onset, and precursor release, rather than causing superficial material loss alone [32]. This conceptual starting point is essential for understanding why relatively low-density infestations may still produce disproportionate quality consequences in Daqu and in downstream Baijiu fermentation.

## 5. Sources of Ecological Disturbances

**Figure 2:**
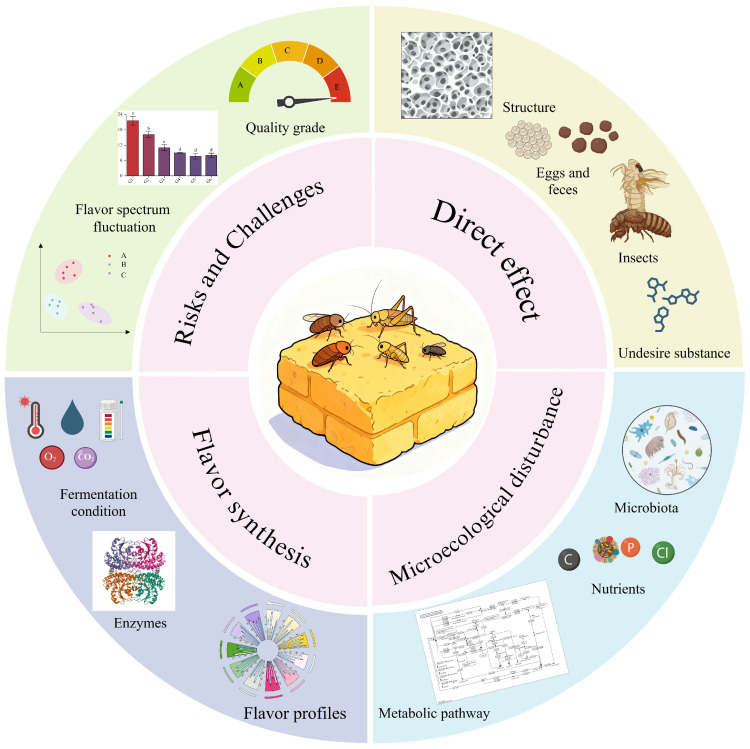
Impact of Daqu-associated pests on the microecology and production of Daqu. Insect feeding, oviposition, feces, exuviae, and other residues can damage the Daqu structure and introduce contamination, thereby disturbing microbiota, nutrient availability, and metabolic pathways. These changes may further affect fermentation conditions, enzyme performance, and flavor profiles, ultimately leading to flavor fluctuation, unstable quality grading, and broader production challenges. Overall, the figure illustrates a directional pathway from insect-induced structural disturbance to microecological imbalance and product-quality variation.

To avoid overstating conclusions, this section explicitly distinguishes direct evidence from Daqu/Baijiu systems, indirect evidence from stored-grain and food-storage studies, and conceptual hypotheses that remain to be validated. As shown in Figure 2, pest-associated ecological disturbance can therefore be discussed at three levels, namely direct interference with the Daqu matrix, indirect microecological risks inferred from related postharvest systems, and possible downstream consequences for flavor formation.

### 5.1. Direct Evidence from Daqu/Baijiu Systems and Current Boundaries

Direct evidence specific to Daqu/Baijiu does not yet demonstrate a fixed causal chain from pest occurrence to a defined flavor defect. What is directly established is that Daqu is an open, non-sterile solid-state starter whose quality depends on stable community assembly, enzyme activity, and the physicochemical environment of the brick [63,90,91,92]. Studies on Daqu microbiology and flavor have further shown that changes in community structure are closely associated with enzyme expression and with the formation of volatile compounds, indicating that disturbance of the solid carrier can have process-level consequences [6,39,42,93].

Accordingly, visible infestation in bricks, fines, stack gaps, or storage areas should first be interpreted as a direct event of physical contamination and process interference. In stored-grain systems, insect damage has been associated with more broken kernels, altered local humidity, and conditions favorable to mold development [94]. For Daqu, the concern is amplified because microbial colonization, heat and mass transfer, and enzyme initiation rely on the structural integrity of a porous solid matrix. Pest feeding, movement, and aggregation may enlarge fissures, increase loose particles, and weaken the carrier that supports early saccharification and fermentation rather than causing merely superficial damage [95]. Once the matrix is destabilized, local ventilation, aeration, and moisture migration can be altered, and the conditions required for timely enzyme initiation may be weakened or delayed [91]. Even so, quantitative thresholds linking infestation density to losses in Daqu functionality or to specific liquor flavor deviations remain insufficiently established [91,96,97].

### 5.2. Indirect Evidence from Stored Grain and Food Storage Systems

Stronger statements regarding hygiene risk, allergen exposure, microbial vectoring, and odor disturbance are presently supported mainly by indirect evidence from stored grain, flour, and food-processing environments rather than by dedicated Daqu studies. In infested grain systems, higher loads of insect fragments, frass, uric acid, and associated microbial contamination have been reported [95,98]. Food and occupational studies likewise show that insect-derived materials, including fragments and dried fecal matter, can contribute to allergic or respiratory reactions, which supports their relevance to sanitation management and worker exposure in enclosed production spaces [58,59,65].

Indirect evidence also supports the possibility that pests can transport microorganisms and intensify local contamination pressure. Studies in stored products report that insects may carry fungal spores, bacterial consortia, or mycelial fragments, including taxa such as *Aspergillus*, *Fusarium*, and *Penicillium* [99]. As insects move across surfaces, dust, and damaged particles, introduced microorganisms can accumulate in cracks and settled microzones, creating joint insect–microbe patches that favor localized ecological change [99,100,101,102,103]. In parallel, stored-product systems have shown that insect infestation may co-occur with higher mycotoxin risk under favorable microclimatic conditions [104,105]. These observations are informative for Daqu risk assessment, but they should be interpreted as analogical support rather than as direct evidence that the same processes occur to the same extent in Baijiu starters.

Evidence for odor alteration is also largely indirect. In grain and flour systems, infestation has been associated with shifts in lipids, proteins, enzyme activities, and volatile profiles, indicating that insect activity can influence aroma-related chemistry [63,90,106]. On this basis, insect secretions, metabolic wastes, frass odors, and decaying remains should be regarded as plausible sources of odor interference in Daqu. However, whether such inputs measurably alter the baseline aroma of Daqu or the final flavor profile of Baijiu still requires direct experimental verification.

### 5.3. Mechanisms of Insect–Microbe–Substrate Interactions

Given the current evidence base, the three-pathway framework discussed below should be regarded as a conceptual hypothesis for future validation rather than as an established causal model. Its scientific rationale is nonetheless grounded in two well-supported premises. Daqu quality depends on microbial succession within an open solid-state system [63,90,91,92], and microbial community structure is closely linked to enzyme activity and flavor generation [6,39,42,93,107,108].

The first proposed pathway is microbial introduction and redistribution. If pests enter during shaping, stacking, turning, room exit, or storage, they may carry exogenous microorganisms into fissures, margins, and dust-rich sites, thereby perturbing the initial microbiome and its subsequent assembly sequence [99]. The second pathway is substrate perturbation. Frass, exuviae, carcasses, fragments, and damaged grain particles can act as localized nutrient pulses. In stored-product ecology, insect residues and necromass are known to reshape carbon and nitrogen flows and to influence microbial substrate utilization [102,109,110]. A related, but still unverified, extension in Daqu is that gut-associated microorganisms or insect-transformed excreta may further modify local substrate chemistry and microbial interactions [110,111,112]. The third pathway is microenvironmental remodeling. Pest activity may enlarge cracks, redistribute fines, and alter local retention of moisture and air exchange, thereby changing pH, water activity, oxygen diffusion, and interfacial contact between substrate particles and microbial films [111,112].

If these three processes operate together, they could plausibly change the timing and proportion of metabolite formation by functional consortia, leading to shifts in esters, alcohols, acids, pyrazines, carbonyls, and related compounds [109,113,114]. At present, however, it is more rigorous to state that pest disturbance may increase the probability of pathway deviation and flavor imbalance than to claim direct formation of any specific off-note compound. Even parallels from other fermented foods, such as mite-associated flavor alteration reported in cheese, should therefore be cited only as conceptual analogies rather than as evidence for Daqu itself [115,116]. Accordingly, possible deviations in esters, aldehydes, pyrazines, amino acid derivatives, or sulfur-containing compounds should be discussed as hypothesis-driven targets for verification, not as already demonstrated outcomes in Daqu [60,63,90,117,118].

### 5.4. Impacts on Fermentation Performance and Flavor

From an industrial perspective, the most defensible conclusion at present is that pest occurrence in Daqu production should be treated as a combined sanitation, process-stability, and quality-risk signal. Indirect evidence from stored-food systems supports concern over impurity accumulation and consumer or hygiene risk [94,119], volatile-profile disturbance and batch instability [95,120], and measurable economic loss under inadequate control [95,121]. For Baijiu production, such risks are more likely to appear first as weakened process robustness and greater batch-to-batch variability than as a single uniform defect aroma [41].

Accordingly, pest management should be integrated into quality assurance rather than handled only as routine housekeeping. Preventive control should emphasize raw-material hygiene, ventilation, dust control, residue removal, and crack management. When infestations are detected, intervention should be followed by verification at the levels of Daqu quality, microbial status, and early precursor or volatile signals. At the same time, an important caveat must remain explicit throughout the manuscript. Most current evidence still derives from grain storage, packaged foods, and general postharvest ecology rather than from the solid-state Daqu system, whose high-temperature, porous, and enzyme-coupled characteristics may alter routes of spread and impact [14,59,122]. Future research should therefore move from analogy to validation by quantifying the relationships among colonization density, residue burden, enzyme capacity, community succession, and flavor deviation under actual Daqu production conditions.

## 6. Control Strategies and Process Management

### 6.1. Environmental and Process Prevention

The topic was framed within a modern IPM perspective, and a low-infestation baseline was treated as the primary goal [123,124]. The first step was to build a suppressive environment for the koji rooms and bricks rather than to rely on reactive measures after outbreaks [123,124]. In solid-state Daqu production, prevention included clean raw and auxiliary materials, ventilation and drying in the koji room, sealing and zoned sanitation, and optimized material turnover. Only the combination of these actions established an effective barrier [123,124].

At the source, grain materials such as wheat, barley, or legumes that carried eggs or residues already acted as a seed bank for pests. Studies in grain science showed that cleaning, sieving, removal of defective kernels, and dust separation reduced early infestation rates by stored-product insects [125]. The same logic applied to Daqu. Removal of impurities and dust before moistening, followed by timely drying, lowered the chance that sources were carried into the koji room and colonized the bricks at the formation stage. After arrival, raw materials were processed without long storage. Prolonged holding increased small humidity and temperature drifts, dust accumulation, and the probability of immigration. Ventilation, humidity, and temperature control, and effective sealing of the qu-room were critical [126]. Proper ventilation allowed rapid moisture loss and cooling in early stacking. Prolonged warm and wet pockets created ideal sites for colonization and spread. Surveys in stored-product systems showed that poorly ventilated corners, wall gaps, and hidden crevices formed aggregation hot spots [99,121,127]. If stacking created heat retention or slow rewetting, preferred niches could form on brick surfaces and edges. Whole-room ventilation, tiered turning, and gap sealing were recommended to avoid leak points and dust traps.

Zoned sanitation was also essential. Qu-rooms were divided into clean areas, operation areas, and stacking areas. Equipment, corridors, and tools were dedicated or cleaned on a routine schedule [128,129]. Dust, fines, and spills were primary early aggregation points. Research showed a strong positive association between dust load and initial colonization [121,125]. After each turning step, accumulated dust and fines were removed. Brick stacks were kept away from walls and corridors, excessive stack height was avoided, bottom ventilation was maintained, and floors were kept clean. Material turnover was optimized as well. Long residence of incoming grain, extended stacking time, and improper post-room aging created retention nodes. These nodes often coincided with humidity rebound, dust buildup, and attractive conditions for pests. Work in storage and processing plants indicated that higher movement frequency and timely turning reduced outbreak risk [99,127,130]. Time limits were defined for each node from intake to Daqu making to exit from the room, with records for traceability. After exit, bricks entered storage or transfer quickly rather than remaining in warm rooms where hidden explosions could start.

A critical view was necessary when translating storage practices to Daqu. Stacks in qu-rooms were irregular, with fast humidity and temperature dynamics and complex structures [10]. Static storage benchmarks such as moisture below thirteen percent or temperatures below fifteen degrees Celsius were not directly applicable [131,132]. Community activity and enzyme dynamics were high. These features favored flavor but also supported rapid pest growth. Preventive control, therefore, required dynamic adaptation rather than static thresholds. Trade-offs were common. Faster drying or earlier turning could protect against pests but might compromise sensitive aroma notes. Preventive design was tuned under flavor constraints rather than as a stand-alone hygiene task [133,134,135].

In summary, environmental and process prevention held strategic value for the aim of controlling pests without loss of aroma. Clean inputs, ventilated and dry rooms, zoned sanitation, and controlled turnover built a low-risk baseline. These steps protected enzyme potential, microbial stability, and flavor consistency at the same time. Mastery of dynamic control of gradients of temperature and humidity, cleaning frequency for dust and fines, ventilation efficiency, turning cadence, and turnover time embeds prevention within the production system rather than as an afterthought.

### 6.2. Physical and Thermal Treatments

Physical tools such as heat, low temperature, or modified atmospheres and inert powders provided gentle choices [41]. These tools minimized chemical residues and protected the flavor-relevant microecology, yet they still faced constraints from brick tolerance, enzyme sensitivity, and stability of flavor precursors [136].

Heat treatment has wide use in stored-product IPM. In practice, facilities were heated to fifty to sixty degrees Celsius and held for hours to ensure lethality across life stages. Reports showed that six to twenty-four hours in the range of fifty to sixty degrees Celsius controlled most stored-product insects [59,121]. In Daqu, this step could be placed near the brick exit or during handling to create a clean window before spreading. Care was needed. Enzyme systems, key precursors, and microbial activity could be temperature sensitive. Heat applied during enzyme initiation or early community assembly might suppress activity and weaken flavor development [121,127,137,138]. Parameters were therefore optimized for temperature and time to achieve lethality with minimal impact.

Low temperature and modified atmospheres offered another path. Reduction in temperature, oxygen depletion, or elevation of carbon dioxide suppressed pest metabolism [139]. Studies reported that more than sixty percent carbon dioxide at thirty degrees Celsius achieved complete adult mortality of several species within four days [140]. Such green methods were attractive due to low residue. In Daqu, bricks could be held under cool or modified atmospheres after exit and before storage to target eggs or larvae. The challenge lay in the brick as a porous body. Water content, pore structure, and gas diffusion complicated the maintenance of target atmospheres and the protection of enzyme and microbial functions. Gas-tight structures, monitoring, and careful matching to process windows were required [141,142,143,144]. Costs, sealing, and integration with turning and ventilation were also addressed.

Inert powders such as diatomaceous earth and zeolitic materials acted as physical barriers. Work showed that some powders at a dose of 0.5 g per square meter caused full adult mortality of three key stored-product species within seven days [145]. The action depended on abrasion of the cuticular wax layer and rapid desiccation rather than toxicity. In Daqu, powders were applied to walls, floors, ducts, and crevices to block ingress. High humidity and heavy dust loads in koji rooms could coat or deactivate powders and reduce efficacy. Mixing into bricks was avoided because pore structures and surface dust content might be altered, and indirect effects on flavor could follow. Doses and locations were chosen with ventilation, cleaning, and turning schedules in mind [72,142].

Operational guidance followed from the goal of control of pests without loss of aroma. First, physical tools were applied before full activation of enzyme systems and before extensive microbial assembly [128]. Second, parameters for temperature, atmosphere, and powders were set by brick tolerance. Pilot tests measured effects on amylase and protease activity, microbial communities, and precursor release before plant-wide adoption. Third, measures were integrated with monitoring, sanitation, ventilation, and turning, not used in isolation. For example, turning was paired with powder sweeping on paths and with a short period of cool or high carbon dioxide holding. Fourth, physical options were prioritized ahead of chemical measures to preserve flexibility for emergency use [121,136,138,140,141,142].

Daqu bricks expanded and contracted with heat and cold, breathed through complex pores, and carried sensitive enzymes. Simple transfer of grain-storage recipes ignored the balance between flavor generation, microbial stability, and the process path of temperature and humidity. Heat left no chemical residue, yet could alter moisture and deactivate enzymes. Low temperature or modified atmospheres were gentle but required sound containment and monitoring to avoid retardation of fermentation onset [146]. Plant trials and risk assessments were therefore essential before scale-up.

In conclusion, physical and thermal tools formed the preferred non-chemical options. Heat offered rapid action. Cool or modified atmospheres offered gentle suppression. Inert powders built peripheral barriers. Successful use depended on process matching. Protection of brick tolerance, microecology, and the flavor pathway was treated as the central constraint.

### 6.3. Irradiation and Electron Beam Technology

Electron beam irradiation was evaluated as a non-thermal option. It delivered a short processing time, high efficacy, and controllable parameters. It still required careful alignment with brick tolerance, flavor sensitivity, and plant logistics. Electron beams, as ionizing radiation, break nucleic acids of pests or microbes. In grain and feed studies, doses near two to five kGy reduced molds, yeasts, and aerobic bacteria with limited impact on nutrition and sensory quality [147,148]. For stored-product pests, the electron beam offered very short exposures, little residue, and no heating. These features positioned the tool as a green option [149,150].

Several advantages were noted for Daqu. Processing time was brief. Thermal disturbance was minimal. Dose, speed, and penetration were adjustable. These traits allowed a node such as brick exit to be coupled to an irradiation pass and then to storage. Yet key questions remained. Brick tolerance had to be proven. Daqu production involved self-heating, active microbial assembly, enzyme initiation, and aroma precursor formation. If irradiation were applied during early biochemical windows, enzyme activation, community assembly, or precursor release might be altered. A dose of three kGy produced little sensory change in some feed models, but those matrices differed from porous and enzyme-rich bricks. Pilot verification of dose against flavor was required to define the minimum effective level, the best timing, and the envelope of effects on key activities and metabolites [151].

Process matching was another challenge. Equipment layouts, penetration through stacked bricks, avoidance of shadowing, immediate transfer to storage, and prevention of re-infestation had to be engineered. Safety, dosimetry, and certification systems were established for plant use. Capital costs and retrofits were justified by risk reduction and quality benefits. Public sources on full-scale use in solid-state starter bricks were still limited, which motivated staged trials. Small sets of bricks were treated at one, three, and five kGy, followed by enzyme assays, precursor release tests, microbial profiling, and final fermentation outcomes.

From a flavor-risk view, the electron beam was gentle but not zero-impact. Some food systems showed small shifts in volatile profiles, oxidation reactions, or color after irradiation [12]. In Daqu, attention focused on the initiation of enzymatic activity, the release of aroma precursors, and the synthesis of aroma compounds. Slight overdoses might suppress sensitive microbes or enzymes and shift esters, aldehydes, pyrazines, and related targets [123]. The tool was therefore embedded within a flavor-risk framework.

Implementation advice followed. The electron beam was positioned as a preferred non-thermal physical option that preceded chemical measures. Design considered process compatibility, flavor sensitivity, operating cost, layout, dose response, and feedback. Pilot verification established dose bands such as two, three, and five kGy and documented effects on enzyme and aroma metrics. Process nodes were synchronized. For example, bricks passed through the beam after exit and before storage, with checks of humidity, microbes, and key precursors. Monitoring confirmed freedom from survivors and compliance of enzyme and aroma metrics [123,147,148,149].

Overall, electron beam irradiation showed promise but required careful alignment with the process. It was accepted as gentle and efficient. It still demanded dose-flavor validation, integration with handling, and continuous monitoring before it could serve the aim of control of pests without loss of aroma.

### 6.4. Biological Control and Ecological Modulation

Entomopathogenic fungi such as *Beauveria bassiana* and *Metarhizium anisopliae* have accumulated evidence in stored-product systems. These fungi attached to the cuticle, penetrated, proliferated, and released toxins. Trials reported that at 1 × 10^8^ conidia per milliliter, mortality of *Sitophilus oryzae* approached ninety percent at twenty degrees Celsius within seven days when *Metarhizium* or *Isaria* strains were used [124,152,153,154]. Such data supported the concept that early deployment in Daqu could depress populations to low baselines. Advantages included low environmental residue and acceptable safety profiles, which fit IPM. Compatibility with the brick microecology remained a key concern [153,155]. The qu-room was hot and humid with complex aeration. Microbial assembly and enzyme activation were rapid. Poor timing or excessive doses could suppress beneficial fungi, yeasts, or actinomycetes and alter amylase, protease, and esterase trajectories. The efficacy of many strains also depended on temperature, humidity, and substrate. Higher temperatures sometimes reduced performance [154,156,157]. Windows for use were chosen before widespread use and during favorable microclimates.

Parasitoids and plant-derived agents added options. *Anisopteromalus calandrae* parasitized larvae or pupae of grain pests and reduced adult emergence in storage systems [158,159]. Plant-based products showed low toxicity, small residues, and potential compatibility with sensitive microecologies, although evidence in storage insects was still developing. Layering these tools created multi-point suppression. Pathogenic fungi reduced adults and larvae. Parasitoids suppressed the next generation. Plant agents protected entry routes and contact surfaces [160].

Operational guidance emphasized staged use. Fungi were applied near intake or early stacking as a base barrier in low-density conditions. Parasitoids or plant agents were placed at vents, wall gaps, and dust-prone corners to weaken ingress. Monitoring tracked mycosis rates, parasitism, and efficacy of plant agents together with community metrics in bricks such as diversity, counts of functional groups, enzyme onset, and aroma outcomes [161,162]. If fungal or plant tools depressed pests but altered enzyme or aroma metrics, timing or dose was adjusted, or gentler strains or formulations were selected. Practical challenges persisted. Performance depended on microclimate, substrate, and density. Costs, stability, and ease of use needed improvement for large plants. Biological tools were slower to act than heat or chemicals, which tested the cadence of production [163,164,165].

In sum, biological control and ecological modulation were integrated with environmental prevention to deliver multi-point suppression. Compatibility with the microecology, enzyme initiation, and aroma formation was verified before scale-up. Future work focused on coupled measurements of pest density, functional microbes, enzyme activity, and aroma compounds inside the bricks to convert the concept of control of pests without loss of aroma into an operational biotechnological program [124,147,154,155,163,164,165].

### 6.5. Boundaries for Chemical Control

Chemical tools were treated as last-resort options in koji and fermentation systems and not as defaults [166,167]. Daqu environments were hot and porous with active microecology. Fumigants, residual sprays, and protectants posed risks to communities, enzyme onset, and aroma formation. Health and environmental concerns were well documented in storage and processing contexts. Phosphine and other fumigants created acute and chronic risks when combined with enclosed spaces, poor aeration, heat, and dust [59,168,169]. Broader reviews described persistence, bioaccumulation, resistance, and impacts on soil, water, plants, and human health [170]. In Daqu, such effects translated into trade-offs. A short-term knockdown could leave residues or depress the activity of fungi, yeasts, or actinomycetes. This depression could reduce the formation of esters, aldehydes, alcohols, and related aroma compounds. Chemical actions also required shutdowns for ventilation and sanitation and disrupted production rhythm.

Within the framework of control of pests without loss of aroma, strict boundaries were set. Non-chemical options were prioritized. Chemical use was limited to emergencies in which other options failed and risk had escalated, and only under quantified and traceable conditions [162]. The scope of use excluded critical windows such as enzyme onset and early community assembly. Small-scale verification preceded plant use. Post-use cleaning and verification included residue tests, monitoring of microbial recovery, and ventilation to restore conditions. Records linked chemical use to flavor and batch outcomes to enable review. Rising resistance to fumigants and protectants, costs, and consumer concern about residues and sustainability further narrowed long-term suitability [133,171,172,173]. Hidden costs included flavor imbalance and loss of product consistency [121,157].

Therefore, chemical control occupied a tightly bound position. It was not routine. It was used only when non-chemical routes were ineffective and after evaluation of risks to microecology and flavor.

### 6.6. Closed Loop of Monitoring, Intervention, and Verification

Modern practice favored a closed loop. Monitoring captured signals, interventions were triggered by thresholds, and verification measured outcomes. In solid-state Daqu production, this loop extended from pests to microecology, metabolism, and flavor [174,175].

Monitoring used pheromone traps, light traps, and quantitative sampling to obtain high-frequency spatial data. Visual checks alone were insufficient. Devices and cards were placed at key positions, and counts were recorded with time and location. Such tools mapped hot spots and movement in storage systems [161,174]. Data were linked to process variables. Examples included stack residence time, number of turns, surface temperature, humidity of bricks, and dust accumulation. Trends were reviewed on a routine schedule. If capture counts rose above predefined bands or if aggregation persisted, the system moved to intervention. Threshold-based control replaced ad hoc reactions [176,177,178].

Triggers were clear and tiered. When counts exceeded baselines by a set percentage or when hot spots persisted, the least intensive tool was used first. Turning, gap cleaning, and targeted trapping were preferred. If signals continued to rise, physical heat or low-dose measures were applied. Actions were traceable and recorded. Advanced setups linked interventions to process analytics and PAT platforms for synchronization [104,175,178].

Verification closed the loop. HACCP identified critical points such as brick exit, early storage, and early turning, and defined monitoring and documentation [179]. PAT tools followed microbial indicators, key aroma compounds, and timing of enzyme onset to quantify the effects of interventions [104,180]. If pests declined while flavor or enzyme metrics shifted, the plan was adjusted. If pests persisted, thresholds and tools were recalibrated.

Embedding the loop within the quality system moved pest control from a stand-alone task to an integrated assurance function. Plants adapt in real time while preserving the flavor pathway. Evidence in storage and food processing was stronger than in solid-state starters, so plants calibrated thresholds and indicators with internal data and trials. The direction was clear. Only through a monitored trigger and verification loop could control of pests without loss of aroma be delivered with consistency.

## 7. Process, Practice, and Enterprise Application for Control of Pests Without Loss of Aroma

Before implementation at the plant scale, a baseline diagnosis was required. This step served as the entry point of pest management and turned the risks across pests, microbes, and aromas into measurable items. Enterprises first mapped risks across Daqu rooms, warehouses, and packaging areas. The map specified how each space (raw material receiving, stacking in the Daqu room, post-room storage, and packaging corridors) aligned with points of pest ingress, microbially sensitive zones, and aroma-critical nodes. For example, traps were placed at the receiving end. Higher trap counts were often recorded along wall cracks and dust accumulations near the Daqu room. Stored-product monitoring recommended at least biweekly quantitative sampling of insects or trap counts to obtain a time-series trend. Next, indicators including trap counts, dust accumulation rate, crack growth, moisture readings, and ventilation temperature–humidity differentials were associated with microbial parameters and key aroma compounds (for example, ethyl acetate, phenylacetaldehyde, and 2,5-dimethylpyrazine) as critical quality attributes. Community assembly shifts were tracked by the proportions of yeasts, filamentous fungi, and actinomycetes at Daqu release as CQAs [46,181,182,183]. Historical data and field surveys then supported threshold setting. Examples included a trap count that exceeded the baseline mean by 1.5 times on two consecutive checks or a crack width above 3 mm with dust thickness above 2 mm. With this baseline, decisions moved from experience to data. A risk model that coupled pest, microbe, and aroma guided later interventions and verification.

The second step was minimal intervention. After diagnosis, when pest or risk indicators reached set thresholds, environmental and physical measures were prioritized over chemical or high-intensity actions. For instance, when edge traps around a fresh batch showed a twofold rise over baseline, the plant initiated restacking, sealing of edges, dust removal, and stronger ventilation, along with inert powders or physical traps at cracks. If two subsequent checks still showed elevated counts, gentle options such as e-beam or mild heat were considered. Chemical treatment remained the final resort. This sequence minimized disturbance to the Daqu microbiome and aroma formation. Teams recorded timing, method, cost, and initial effects to enable later optimization. The QbD and PAT principle of minimum effective intervention was followed. During environmental optimization, ventilation and humidity changes, dust reduction, and moisture decline were monitored to ensure that actions targeted pest suppression rather than surface housekeeping only [184].

The third step was evidence closure. After interventions, verification confirmed control efficacy and integrated the results into quality assurance. HACCP and QbD PAT served as the reference framework [185]. Key control points were defined at re-stacking in the Daqu room, post-room stacking, and pest monitoring. Each batch recorded trap counts, crack depth, dust mass, stack temperature–humidity differentials, and airflow, with alert thresholds. After release or storage entry, samples were tested for microbial communities by 16S and ITS sequencing to obtain yeast or mold proportions, for key volatiles such as ethyl acetate and phenylacetaldehyde, and for sensory outcomes. Online and offline PAT tools, such as near-infrared, e-nose, and real-time volatile monitoring, created a data stream to connect process shifts with microbial and metabolic responses [186,187]. If verification showed pest control but a deviating volatile profile or delayed enzyme activation, the model parameters and trigger thresholds were adjusted, or protective measures for the microbiome were revised. This formed a closed loop from diagnosis to intervention to verification and ensured low pest risk, together with aroma consistency and fermentation performance.

In enterprise deployment, the three steps were executed through an information backbone, decision rules, and an operations team. In practice, a monitoring platform was established with traps, regular sampling, dust and fines measurement, temperature–humidity sensors, and crack imaging tools. Computer vision models such as yo-LO SG Insects were used for assisted recognition [188]. Monitoring data were linked to the process system with dashboards, early-warning thresholds, and named owners. When a trigger was reached, production, quality, and safety units acted together. The execution team performed restacking, cleaning, ventilation, and sealing while keeping gentle options such as e-beam as reserves. During verification, online volatile monitoring, periodic microbiome analysis, and sensory sampling were integrated into batch reports and fed back into the quality system. Several challenges were recognized. Baseline diagnosis required early investment. Many plants lacked online sensors, trap networks, or PAT infrastructure [174,177,189]. The promise of control without loss of aroma set a high bar for consistency. Without sound verification and feedback, plants could achieve low pest counts yet still face aroma drift. A phased pilot on one line or one room for three to six months was therefore recommended before scale-up. Overall, the three-step pathway provided a system framework that joined pest monitoring, trigger-based intervention, and aroma assurance. Through risk mapping, minimum interventions, and HACCP plus QbD PAT verification, enterprises built a closed loop from pest surveillance to aroma quality.

## 8. Prospects and Conclusions

### 8.1. Future Research Directions

Current evidence supports treating insects in Daqu production as a potential source of ecological disturbance rather than as a confirmed direct cause of specific flavor defects. Because Daqu production depends on natural inoculation, open fermentation, and microbiota-driven enzyme and flavor formation, future work should focus on whether insect occurrence measurably alters microbial succession, key enzyme activities, and early flavor precursors under actual Daqu conditions [6,30]. Multi-omics approaches, including 16S/ITS sequencing, metagenomics, metatranscriptomics, metabolomics, and volatile profiling, may provide a practical route for testing these links in a quantitative manner [190].

### 8.2. A Conceptual Framework Requiring Validation

**Figure 3:**
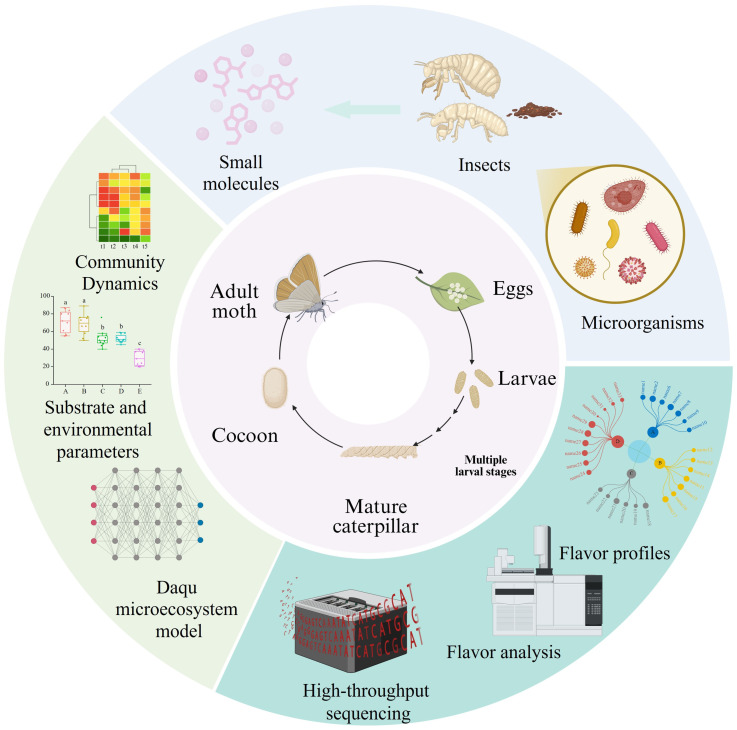
Cross-domain coupling among Daqu-associated pests, microorganisms, and flavor. The central circle represents the continuous pest life cycle, indicating that all developmental stages may act as recurrent disturbance sources during Daqu production and storage. The upper-right sector shows insect–microbe–metabolite interactions, highlighting pests as both substrate consumers and potential vectors or modifiers of microbial communities. The lower-right sector indicates that such disturbances may alter flavor profiles and can be monitored by high-throughput sequencing and flavor analysis. The left sector represents the system-level feedback layer linking community dynamics, environmental factors, and the Daqu microecosystem model. Overall, the figure illustrates a directional pathway from pest persistence to microbial and metabolic perturbation, flavor variation, and mechanistic interpretation for process control.

At present, the proposed insect-mediated effects should be regarded as a conceptual framework for future validation rather than as an established mechanism. Three pathways remain especially worthy of investigation: microbial vectoring by insect bodies or residues, local substrate perturbation caused by frass, exuviae, and necromass, and gut-mediated biotransformation of returned particles [41]. These routes are biologically plausible and are broadly consistent with indirect evidence from stored-product systems, where insect infestation has been associated with volatile changes and quality deterioration (Figure 3). However, whether these pathways occur in Daqu and whether they are sufficient to alter Baijiu flavor remains to be demonstrated experimentally.

## 9. Limitations

This review has several limitations. First, direct evidence from Daqu and Baijiu systems remains limited, and many mechanistic interpretations are inferred from stored grain, food storage ecology, or general pest-management literature. Second, the specific features of Daqu, including open fermentation, high-temperature phases, porous solid matrices, and complex microbial succession, mean that findings from other systems cannot be transferred directly. Third, the proposed three-pathway hypothesis should be understood as a research framework rather than a confirmed causal model. Accordingly, the present article is best interpreted as a synthesis of direct evidence, indirect evidence, and conceptual hypotheses with different levels of certainty.

## 10. Conclusions

As summarized in Table 2, Daqu-associated insects should be regarded as a potential ecological disturbance factor relevant to process stability rather than as a minor sanitation issue alone. Current Daqu research clearly supports that Daqu is an open, microbially driven fermentation starter and that microbial succession is closely linked to enzyme activity and flavor formation. However, most insect-related mechanisms in Daqu are still not directly validated, and the proposed three-pathway framework should therefore be treated as a conceptual model for future testing rather than as an established causal mechanism. From a practical perspective, insect management should be integrated into quality assurance through prevention, monitoring, threshold-based intervention, and post-control verification so that pest control supports flavor stability without causing unnecessary interference with Daqu microecology.

## Figures and Tables

**Table 2:** Integrated summary of evidence levels, proposed mechanisms, control implications, and research priorities for Daqu-associated insects in Baijiu production.

Domain	Core Point	Direct Evidence from Daqu/Baijiu Systems	Indirect Evidence or Conceptual Inference from Related Systems	Potential Impact on Daqu and Baijiu Quality	Control or Monitoring Implication	Research Priority
Insect occurrence and process relevance	Insects should be treated as process-relevant ecological disturbance factors rather than as marginal sanitation issues only	Surveys, workshop observations, and process-based discussions in the reviewed manuscript indicate that insects can occur during brick making, stacking, fermentation, storage, and workshop management	Related stored-product studies show that infestation is common in solid food matrices and can affect quality, storage stability, and processing performance	Increased process instability, higher risk of local contamination, and greater uncertainty in flavor consistency	Include insect occurrence in the routine process risk assessment rather than limiting it to sanitation inspection	Establish plant-specific occurrence maps by stage, location, and season
Structural disturbance of the Daqu matrix	Feeding, crawling, and residue accumulation may alter fissures, porosity, dust distribution, and local moisture migration	Daqu studies support the importance of matrix integrity, porosity, temperature, humidity, and microbial colonization for proper community assembly and enzyme formation	Stored-grain studies indicate that insect damage increases broken material, local humidity variation, and structural deterioration	Disturbance of the physical carrier that supports microbial colonization, heat and mass transfer, and enzyme initiation	Monitor crack ratio, dust deposition, edge damage, and local moisture retention in infested zones	Quantify links between infestation intensity and changes in Daqu physicochemical structure
Hygiene, impurity, and allergen burden	Fragments, exuviae, carcasses, and feces may increase impurity load and sanitation pressure	Direct Daqu-specific quantification remains limited	Food-processing and stored-product studies show associations with insect fragments, fecal material, microbial burden, and occupational exposure risks	Lower hygienic quality, possible increase in biological load, higher plant sanitation, and audit risk	Add impurity-related indicators and residue checks into workshop hygiene control	Define acceptable residue thresholds for Daqu production scenarios
Microbial vectoring pathway	Insects may carry exogenous or environmental microbes into bricks, fissures, and stack gaps	Daqu research clearly shows that microbial succession is central to enzyme activity and flavor formation, but direct demonstration of insect-mediated inoculation in Daqu is still lacking	Stored-product studies indicate that insects can carry fungal spores, bacterial cells, and mycelial fragments and redistribute them in food environments	Altered initial seeding pattern, shifted succession order, and possible disturbance of dominant functional guilds	Extend pest monitoring from counts alone to possible microbial carriage risk	Characterize insect-surface microbiota and compare it with the early-stage Daqu community assembly
Substrate pulse or necromass pathway	Bodies, exuviae, feces, and damaged particles may create localized nutrient pulses rich in nitrogen, chitin, and lipid residues	Daqu systems support the general importance of substrate state and microbial metabolism, but direct validation of insect-derived nutrient pulses is insufficient	Stored-product ecology suggests that residues and necromass can reshape local nutrient flow and microbial utilization	Selective enrichment or suppression of functional microbes, altered carbon and nitrogen flow, and biased precursor formation	Strengthen crack cleaning, dust removal, and residue management as part of quality control	Measure local chemistry, particle size, nitrogen distribution, and microbial response in residue-rich microzones
Gut-mediated biotransformation pathway	Insects may preprocess substrate particles through gut microbes and digestive enzymes and return transformed particles to the brick	No direct Daqu-specific proof has yet been established	Biologically plausible from insect digestive ecology and gut-microbe studies in grain-feeding insects	Changed accessible substrate spectrum, altered selective pressure, and modified timing of precursor release	Regarding insects, not only as removable contaminants but also as potential agents of substrate pre-transformation	Analyze insect feces chemistry, gut microbiota, and subsequent effects on Daqu enzyme activity and flavor precursors
Microenvironmental remodeling	Insect activity may jointly reshape pH, water activity, oxygen diffusion, fines concentration, and biofilm–substrate interfaces	Daqu’s research strongly supports the importance of environmental parameters for microbial stabilization and flavor-related metabolism	Related systems suggest that infestation can induce local hotspots, moisture accumulation, and altered VOC behavior	Shifts in micro-zonal metabolism, community balance, and enzyme onset timing	Combine insect monitoring with local environmental sensing rather than using insect counts alone	Build coupled models linking insect activity, microenvironmental change, and community drift
Flavor-pathway deviation	Insect disturbance may ultimately affect ester, alcohol, aldehyde, pyrazine, sulfur, and other flavor-related pathways	Daqu and Baijiu studies support the link between microbiota, enzyme activity, and flavor compound formation	Infested flour and cereal studies report detectable VOC differences after infestation, supporting the plausibility of flavor-level effects	Flavor imbalance, weakened style typicality, batch variation, or latent aroma drift	Use early flavor precursors and volatile profiling as verification tools in addition to pest inspection	Verify whether specific volatile shifts can be reproducibly linked to insect density or residue load under Daqu conditions
Integrated pest management	Pest control should prioritize prevention, monitoring, and staged intervention rather than relying on chemicals alone	This aligns with the process-control logic proposed in the manuscript	IPM literature consistently defines prevention, monitoring, and corrective action as the basis of stored-product pest management	Better protection of process stability while minimizing unintended interference with microbiota and aroma formation	Prioritize source reduction, environmental management, physical or thermal suppression, and carefully bounded chemical use	Develop Daqu-adapted IPM criteria balancing pest suppression and flavor preservation
Monitoring–intervention–verification loop	Effective control requires a closed loop from detection to threshold-based action and post-control verification	Strongly consistent with the logic of HACCP, PAT, and quality-by-design style process management proposed in the manuscript	Stored-product IPM also emphasizes monitoring and action thresholds for corrective measures	Improves within-batch and between-batch consistency and converts pest control into a measurable quality-management process	Track insects, residues, cracks, dust, community indicators, enzyme activities, and key volatiles in one loop	Define data-driven thresholds for action and feedback adjustment
Digitalization and industrial deployment	Future implementation should move toward enterprise-level digital warning and rapid response platforms	Direct evidence is still emerging	Current literature supports increasing use of sensing, VOC monitoring, and intelligent management in fermentation and grain systems	Faster warning, more precise intervention, and stronger traceability of quality variation	Integrate trap data, environmental sensing, microbiome indicators, and aroma signals into linked dashboards	Build cross-plant models and validate the transferability of thresholds and early-warning indicators
Evidence boundaries and limitations	The current knowledge base mixes direct evidence, indirect evidence, and conceptual hypotheses	Direct Daqu/Baijiu evidence is strongest for the role of microbiota, process environment, and flavor formation	Many insect-specific mechanisms still rely on analogy from stored grain and food-storage ecology	Risk of overstatement if all mechanisms are treated as already proven in Daqu	Conclusions and management recommendations should be expressed with evidence boundaries should be made explicit	Move from conceptual plausibility to causal validation under real Daqu production conditions

## Data Availability

No new data were created or analyzed in this study. Data sharing is not applicable to this article.

## Supplementary Materials

The following supporting information can be downloaded at: https://www.mdpi.com/article/10.3390/foods15071195/s1, Table S1: Characteristics of the microenvironment in Daqu. References [10,11,30,35,41,53,55] are cited in the supplementary materials.
