# Supplementary material for: Ecological Risks in Daqu Storage and Their Impact on Baijiu Flavor: Precision Process Strategies for Damage Mitigation While Preserving Aroma
Source: Foods. 2026 Apr 2;15(7):1195. doi: 10.3390/foods15071195 (PMC13073634; doi:10.3390/foods15071195)
Supplement: Supplementary file 1 [file foods-15-01195-s001.zip › foods-4211386-supplementary.pdf]

Supplementary materials for

Ecological Risks in Daqu Storage and Their Impact on Baijiu Flavor: Precision Process Strategies for Damage Mitigation while Preserving Aroma

Dandan Song[1,2], Chunlin Zhang [1,2], Yashuai Wu [3], and Liang Yang [1,2,*]
1 School of Brewing Engineering, Moutai Institute, Ren Huai 564501, China; songdd0330@163.com ; zcl818075@163.com ; moutaiyl0725@163.com ;
2 Guizhou Key Laboratory of Microbial Resources Exploration in Fermentation industry, Kweichow Moutai Group, Zunyi 564501, China; songdd0330@163.com; zcl818075@163.com; moutaiyl0725@163.com;
3 School of Food Science and Engineering, South China University of Technology, Guangzhou 510640, China; wyss995418706@163.com ;
* Correspondence: Liang Yang; moutaiyl0725@163.com ;
Address: School of Brewing Engineering, Moutai Institute, Renhuai 564501, China.

Table S1 Characteristics of the microenvironment in Daqu

Type	Fermentation time/d	Temperature/ ℃	Dominant bacteria	Dominant fungi	Characteristics	References
HTD	40	> 60℃	*Bacillus, Kroppenstedtia, Saccharopolyspora, Scopulibacillus, Virgibacillus, Weissella, Thermoactinomyces, Lactobacillus, Oceanobacillus, Streptomyces*	*Thermoascus, Thermomyces, Saccharomycopsis, Pichia, Aspergillus, Rasamsonia*	（1） The daqu-making room features an open structure with high environmental openness； （2） The intensity of volatile signals is strong. （3） A large number of pyrazines and furans are generated through Maillard reaction and other processes. （4） An insect community dominated by heat-tolerant and aroma-philic species (e.g., Araecerus fasciculatus,	[1,2]
MTD	30	50-60℃	*Weissella, Lactobacillus, Pediococcus, Streptococcus, Staphylococcus, Thermoactinomyces, Streptococcus, Bacillus,*	*Lichtheimia, Pichia, Rhizopus, Lichtheimia, Rasamsonia, Byssochlamys, Talaromyces, Penicillium*	（1） Medium environmental openness; （2） Medium intensity of volatile signals; （3） Potential highest species diversity; （4） A mixed insect community consisting of widespread species	[3,4]

			Pseudomonas, *Pediococcus*		and moderately thermophilic species.	
LTD	28	40-50℃	*Bacillus* (*Bacillus subtilis*, *Bacillus pumilus*, *Bacillus licheniformis*), *Lactobacillus* (*Lactobacillus plantarum*, *Lactobacillus curvatus*), *Pediococcus* (*Pediococcus pentosaceus*) *Weissella* (*Weissella cibaria*, *Weissella anomalus*)	*Penicillium*, *Rhizopus* (*Rhizomucor stolonifera*), *Pichia* (*Pichia kudriavzevii*), *Saccharomycopsis* (*Saccharomycopsis fibuligera*), *Wickerhamomyces* (*Wickerhamomyces anomalus*),	（1） The qu-making room is relatively closed with low environmental openness; （2） Weak intensity of volatile signals; （3） Few insect species; （4） Dominated by conventional stored-grain	[5-7]

References

1. Yang, L.; Fan, W.; Xu, Y. Effects of storage period and season on the microecological characteristics of Jiangxiangxing high-temperature Daqu. Food Res. Int. **2024**, 196, 115034.
2. Yang, L.; Fan, W.; Xu, Y. Qu-omics elucidates the formation and spatio-temporal differentiation mechanism underlying the microecology of high temperature Daqu. Food Chem. **2024**, 137988.
3. Shen, S.; Hu, B.; Zheng, J.; Xing, S.; Shi, G.; Zhang, B.; Lin, L.; Zhang, C. Unraveling flavor formation in Jiuqu: Source pathways, influencing factors, and regulatory strategies. Food Chemistry: X. **2025**, 31, 103009.
4. Du, H.; Wang, X.; Zhang, Y.; Xu, Y. Exploring the impacts of raw materials and environments on the microbiota in Chinese Daqu starter. Int. J. Food Microbiol. **2019**, 297, 32-40.
5. Huang, X.; Li, R.; Xu, J.; Kang, J.; Chen, X.; Han, B.; Xue, Y. Integrated multi-omics uncover viruses, active fermenting microbes and their metabolic profiles in the Daqu microbiome. Food Res. Int. **2025**, 116061.
6. Luo, Y.; Wu, L.; Wu, M.; Liao, H.; Yao, Y.; Luo, Y.; Ji, W.; Gao, L.; Wang, Z.; Xia, X. Intelligent Manufacturing Challenges and Directions of the Baijiu Starter Culture-Daqu Industry: Microbiome and Engineering Perspectives. Trends Food Sci. Technol. **2024**, 104724.
7. Hou, Q.; Wang, Y.; Cai, W.; Ni, H.; Zhao, H.; Zhang, Z.; Liu, Z.; Liu, J.; Zhong, J.A.; Guo, Z. Metagenomic and physicochemical analyses reveal microbial community and functional differences between three types of low-temperature Daqu. Food Res. Int. **2022**, 156, 111167.
